# Factors associated with fathers' involvement in infant and young child feeding and nurturing care in the urban slums of Bangladesh: A cross‐sectional study

**DOI:** 10.1002/fsn3.3390

**Published:** 2023-05-06

**Authors:** Dipika Shankar Bhattacharyya, Tonmoy Sarker, Nargis Akter, Sohana Shafique, Mohammad Hayatun Nabi, Mohammad Delwer Hossain Hawlader, Dipak Kumar Mitra

**Affiliations:** ^1^ Health System and Population Studies Division icddr,b Dhaka Bangladesh; ^2^ Department of Public Health North South University Dhaka Bangladesh; ^3^ Infectious Disease Division icddr,b Dhaka Bangladesh

**Keywords:** Bangladesh, fathers' involvement, gender role, infant and young child feeding, nurturing care, nutrition, slum, urban health

## Abstract

Optimal infant and young child feeding (IYCF) and nurturing care during the first years of children's lives are crucial for ensuring their growth and development. The IYCF and nurturing practices are associated with a multifaceted interplay of context‐specific factors where fathers' involvement is necessary. The aim of this study is to explore the current scenario of fathers' involvement in IYCF practices in the urban slums of Bangladesh. A cross‐sectional survey among the residents of the Sat Tola slum in Dhaka, the capital of Bangladesh was conducted. To avoid social desirability bias, 361 mothers of children aged <24 months were interviewed regarding fathers' involvement. An operational definition of father's involvement was developed based on 11 criteria and then scoring was applied to classify ‘Good Involvement’ and associated factors were identified using multivariable logistic regression. Fathers' mean age was around 31 (SD ± 6.09) years and the majority of them (44.32%) completed primary education. Fathers had diversified occupations such as day laborer (32.41%), garment worker (22.71%), and business (14.96%). Factors that were significantly associated with the father's involvement in IYCF practices include educational status (aOR = 3.98, 95% CI = 1.91, 8.26, *p* < .00) and theiroccupational status (aOR = 0.34, 95% CI = 0.16, 0.70, *p* = .00). Fathers were more active for their first child (aOR: 1.99, 95% CI = 1.04, 3.79, *p* = .03). Having child in the age group of 14–20 months (aOR = 2.73, 95% CI = 1.32, 5.64, *p* = <.01) and wife in the age group of 21–30 years (aOR = 2.34, 95% CI = 1.20, 4.58, *p* = .01) were significantly associated. The study finding explored that fathers' education and occupation as well as mothers' age and education were significantly associated factors for fathers' involvement in the IYCF practices. Further longitudinal studies are recommended to establish the causal relationship between fathers' involvement with IYCF and their impact on child growth and development.

## Graphical abstract

Fathers' involvement in infant and young child feeding (IYCF) is important but it is an under‐researched issue specifically in urban slums of Bangladesh. We identified overall that about one‐third of the fathers (63%) were found to have good involvement in IYCF practices. Fathers' occupation and education are significantly associated with fathers' poor involvement in the IYCF practices. Findings could be useful to design context‐specific interventions for addressing the undernutrition problem among children living in the urban slum.

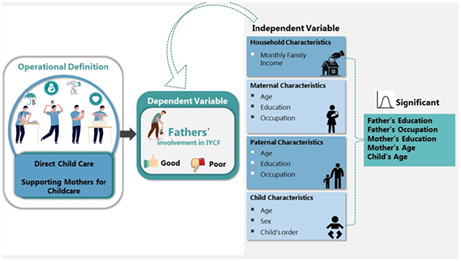

## 1 INTRODUCTION

Ensuring adequate and appropriate nutrition in infancy and early childhood is one of the central prerequisites for the growth and development of children. Poor health among children is significantly associated with the lack of optimum level nutrition during infancy and early childhood. Undernutrition is accountable, directly or indirectly, for half of all under‐5 children deaths around the world (Black et al., 2013; UNICEF & WHO, 2020). Recent systematic reviews suggest that inappropriate childhood nutrition is also linked with other impairments including stunting, physical disability, childhood obesity, and poor intellectual performance–all of which are growing public health concerns in many countries (Hume‐Nixon & Kuper, 2018; Tandon et al., 2016).

To address the problem, the first 2 years of children's life is regarded as a critical window of opportunity since the optimal feeding practice during this time facilitate apposite growth and development (Prentice et al., 2013). Evidence suggests that among the top 15 interventions for preventing under‐5 mortality, the two interventions, namely, exclusive breastfeeding up to 6 months of age and introduction to complementary feeding from 6 months, can prevent almost one‐fifth of under‐five mortality in developing countries (Grantham‐McGregor, 2007).

Optimal infant and young child feeding (IYCF) is, therefore, regarded as one of the vital strategies for preventive health and nutrition interventions in many global recommendations. The World Health Organization (WHO) made a “Comprehensive implementation plan on maternal, infant and young child nutrition,” which has been endorsed in 2012 by the Member States with an aim to promote and support the countries regarding this (McGuire, 2015). In addition to this, there is a separate “Global strategy for infant and young child feeding” uptake by WHO for protecting, promoting, and supporting appropriate IYCF practices across the globe (santé et al., 2003).

In line with these global recommendations, Bangladesh also has taken initiatives in support of IYCF. For example, the National Strategy on IYCF in Bangladesh was developed in 2007. Based on the recommendation of this strategy, an Operational Plan for National Nutrition Services (NNS) (2011–2016) was also formulated (Directorate General of Health Services, 2011; Institute of Public Health Nutrition, 2007). According to the national strategy, the Bangladesh Government recommends several practices for IYCF (Institute of Public Health Nutrition, 2007).

Despite these policy‐level recommendations and initiatives, there are substantial concerns about the nutrition of children in Bangladesh, especially in the urban slum. Although Bangladesh has developed a strong culture of breastfeeding across the country, it has been found from studies that there are inappropriate breastfeeding and complementary feeding practices. According to the latest Bangladesh Urban Health Survey, among the children aged between 6 and 23 months, only one in four of them is receiving the proper IYCF practices in the urban slum areas, which is 40% for nonslums (NIPORT et al., 2015). Moreover, the previous evidences suggest that the rapid urbanization in Bangladesh will result in unprecedented demographic changes as 50% of its population will be living in urban areas by 2050 (Shafique et al., 2018). Most of these urban population growths will be in slums and already 30% of urban populations are living in slum settlements (Adams et al., 2015). Among the urban areas, Dhaka is currently housing 48% of the slum dwellers (Tuthill et al., 2014) which will be expected to be increasing in the future because of the multiple push and pull factors. Therefore, a major portion of infants and young children in the country is living in urban slum areas, which warrants specific attention regarding IYCF and other interventions in order to ensure their optimum nutrition.

In fact, the IYCF practices are associated with multifaceted interplay of context‐specific factors such as the educational status of mothers, socioeconomic profile, feeding‐related beliefs and preferences at the community level, healthcare access through local healthcare workers, media orientation, and little knowledge of optimal feeding behaviors (Haider et al., 2010; Joshi et al., 2012; Ruel et al., 1999; Senarath et al., 2012). Predominantly child health care and nurturing are ‘mother centric’, and the participation of the father is, thus, not emphasized in most low‐income countries (Hackett et al., 2015). However, there are international policy directions that emphasized father's involvement in the IYCF practices. For example, in the International Conference on Population and Development (ICPD), paternal involvement was specifically highlighted for maternal and child care (Tuthill et al., 2014). The evidence suggests that fathers' involvement has a positive and strong association with children's overall nutrition and cognitive skills development (Fan & Chen, 2001; Jeynes, 2007). Previous studies also recommended that in order to improve the IYCF, fathers should be included in the program design with tailored messages (Sanghvi et al., 2013). The fathers' involvement is also encouraged in current policies and guidelines of Bangladesh. However, in the global literature fathers' involvement in the IYCF practices generally lacks appropriate attention. Similarly, the context of Bangladesh is not well documented too. Specifically, a vast majority of urban poor families are recent migrants from rural areas, with a lack of social capital and thus face livelihood challenges to address health and nutrition problems (Adams et al., 2018). Previous studies suggest that fathers' involvement in childcare is largely influenced by the existing gender norms, the father's professional status as the main breadwinner of the family, and mother's engagement in the formal labor market (Raley et al., 2012). However, there is lack of evidence revealing the realities of fathers' involvement and associated factors such as profession, education, etc. in the urban informal settlement from low‐ and middle‐income countries like Bangladesh. From this perspective, this study aims to explore the current scenario of fathers' involvement in IYCF in urban slums of Bangladesh so that context‐specific evidence can be generated regarding the fathers' involvement in order to develop targeted approach for future intervention.

## 2 METHODS

### 2.1 Study design, sites, and population

A descriptive cross‐sectional survey was conducted from September 2020 to February 2021 for collecting data. The study's target population was the dwellers of Sat Tola slum located in the middle of commercial areas named Mohakhali and Tejgaon, at the center of Dhaka, the capital of Bangladesh. The Sat Tola slum was selected as it is one of the largest and most representative slums of Bangladeshi city, where the majority of the residents are involved in economic activities. According to a recent estimate, approximately 50,000 people live in the Sat Tola slum (Lata, 2020).

### 2.2 Sampling and recruitment of study participants

The sample size for this study was determined using single population proportion formula with the assumptions of 95% confidence level, allowable error (0.05), and assuming the proportion of father's involvement in IYCF practice in the urban slum (*p* = .5) as there was no similar study in the same setting. Hence, the total required sample size for the study was 384. So, initially, we recruited a total of 384 participants for the study. However, we dropped partially completed interviews and as a result, we found that a total of 361 respondents completed the full interview which was used in the analysis.

We applied systematic sampling technique for household selection. The first household was randomly chosen from the slum located at the approximate geographical center and then the data collectors proceeded to the next household until the required study participants were sampled. From each household, one participant was selected. Considering the objective of the study, we included the women aged 15–49 years having children aged <24 months of age and the women who are usual residents (living for more than 6 months) in the selected slum. Women with speech and hearing disabilities or severe illness, and who did not agree to participate in the study were excluded from the study. Besides, since the study objective is about the fathers' involvement, we excluded those women who are not living with their husbands currently.

### 2.3 Data collection

We used a structured paper‐based questionnaire to collect data from a team consisting of two data collectors. The study investigators (DSB and NA) provided training to the data collectors about the study objectives, methodology, and on the questionnaire. The training sessions also included sessions about rapport building, ethical issues such as confidentiality, cultural awareness, and risk management including COVID‐19 preventive measures. Once the training was done, the study investigators arranged a pilot survey, and necessary corrections were made following the pilot.

The data were collected through face‐to‐face interviews conducted at the respondent's home maintaining privacy. Before the start of the interview, the data collectors clearly communicated that participation in the study was voluntary and no incentive would be given. The study investigators (NA and DSB) supervised the data collection process and reviewed all the data collection sheets for completeness, accuracy, and consistency. From each respondent, data were collected considering both independent and dependent variables of the study. The independent variables included sociodemographic characteristics of the mother, father, and child; as well as the economic condition of the household. Regarding mother's and father's sociodemographic characteristics, we collected their age, education, and occupation information. For the child, we collected child's age, sex, and birth order data. In addition, to understand the economic condition, we collected monthly household income. For the dependent variable, we asked 11 specific questions to the mother regarding father's involvement in the IYCF practices.

Previous research in a similar country setting (Abera et al., 2017) showed that asking questions to fathers about their involvement in IYCF practices has the potential to bring in the “Social Desirability Bias” since the fathers might have a tendency to report an answer in such a way that they think to get more socially acceptable than what is their “true” answer. In order to avoid the social desirability bias, we directed the questions to mothers only. The questions captured fathers' involvement status in terms of the support they provide to the mothers as well as the direct childcare they do for ensuring the IYCF. Regarding fathers' support, we asked questions on whether the fathers supported mothers on health service utilization, encouraged early initiation of breastfeeding and colostrum feeding, discouraged prelacteal feeding, encouraged breastfeeding on demand, encouraged exclusive breastfeeding for 6 months, and psychologically supported the mothers to ensure appropriate IYCF. We also asked questions on whether the fathers did direct child care, helped mothers by doing other household chores, arranged additional food for mothers, that is, BF mothers, and supported mothers in BF and child feeding in public places. From these 11 structured questions, we introduced a scoring to identify the Good and Poor Involvement of the fathers. For each “Yes” answer, 1 point was given while 0 was assigned for each “No” answer from the respondent. “Good involvement” was defined if the father scored 6 or more, while “poor involvement” was defined as a score less than 6. These criteria have been selected based on a previous study that explored father's involvement conducted in a similar country setting (Abera et al., 2017). While scoring the father's involvement, we did not separate father's direct childcare issues from father's support to the mother considering previous evidence that father's involvement is a comprehensive construct that includes direct father–child engagement as well as fathers' interaction with other household members especially with mother of the child (Sarkadi et al., 2008).

### 2.4 Data management and analysis

We analyzed the collected data to present descriptive statistics as percentage (95% CI) for categorical variables or mean ± SD for continuous variables. Bivariate analysis with Chi‐square test was performed to see the association between fathers' involvement status and the demographic variables. Finally, we performed a multivariable logistic regression model. In the multivariable model, the variables with a *p*‐value < .25 in the bivariate model were included. The backward elimination process was performed to finalize the multivariate model. We used the dichotomized analysis for this study primarily because of the resource and time constraints. Besides, the scope of the research question was limited only to see the existing scenario on fathers' involvement in IYCF in urban slums of Bangladesh. We did not explore further how it is associated with child outcomes in terms of socio‐emotional development and other family dynamics like motherhood–fatherhood adult adjustment dimension. The statistical analysis was carried out by utilizing the computer software Stata (version 13). Data were checked, edited, and coded properly before performing the analysis.

### 2.5 Ethical considerations

For the Institutional Review Board (IRB) approval, the ethical approval was taken from North South University (NSU) ethical review committee. Confidentiality was maintained throughout the research process; the interviews were taken in a venue at the convenience of the interviewee where confidentiality was maintained. Besides, the papers containing the information of the study participants remained to the study investigators in a locked cabinet and no one except the investigators involved with this research was able to look at the interview notes or any other information. It was ensured that identifiable information would not be disclosed without the permission of the respondents. Informed consent of the respondents (mothers) was obtained before the interview. Respondents were ensured that they have the right to decline and withdraw from the study at any time would be accepted. Regarding conflict of interest, the authors disclose no conflict of interest in conducting the study. Informed consent of the respondents (mothers) was obtained before the interview. Respondents were ensured that they have the right to decline and withdraw from the study at any time would be accepted. Regarding conflict of interest, the authors disclose no conflict of interest in conducting the study.

## 3 RESULTS

### 3.1 Demographic characteristics of respondents

A total of 361 mothers who had children aged less than 24 months participated in the study, which gives a response rate of 94.01%, out of 384 mothers attempted. The mean age for the children was 9 (SD ± 6.26) months. Besides, the numbers of male and female children were approximately similar.

The mean age of mothers was found around 25 (SD ± 5.45) years of age. Forty‐seven percent (*n* = 170) mothers got primary education followed by secondary and above education (43%, *n* = 154). Regarding occupation, 89.75% of mothers were found homemakers while around 7% were garment workers followed by day laborer and business.

Fathers' mean age was around 31(SD ± 6.09) years. Like the trend of mother's education, 44.32% of fathers completed primary education (*n* = 160) followed by 41.83% of fathers who attended up to secondary education and above (*n* = 151). Wide variety of professions was observed among fathers as maximum of them were engaged as day laborers (32.41%, *n* = 117), garment workers (22.71%, *n* = 82), and business (14.96%, *n* = 54).

Regarding monthly household income, 34.35% (*n* = 124) reported that their income is within the range of 5000–10,000 BDT ($58–$115; 1USD ~ 87BDT) per month, while 36% (*n* = 130) was within the range of 11,000–15,000 BDT ($127–$173). Table 1 depicts detailed information on the background characteristics of the study respondents.

**TABLE 1:** Descriptive statistics of the socioeconomic characteristics of the study participants.

Variables	Mean	SD	%	*N*
Child's age	8.85	6.26	–	361
Fathers' age	30.75	6.09	–	361
Mother's age	24.73	5.45	–	
Child's sex				
Male	–	–	48.75	176
Female	–	–	51.25	185
Child's parity				
1st parity	–	–	45.43	164
Other than 1st parity	–	–	54.57	197
Father's education				
No education	–	–	13.85	50
Primary	–	–	44.32	160
Secondary and above	–	–	41.83	151
Father's occupation				
Business	–	–	14.96	54
Day labor	–	–	32.41	117
Driver	–	–	2.77	10
Garments worker	–	–	22.71	82
Service	–	–	8.31	30
Shop	–	–	8.86	32
Unemployed	–	–	2.22	8
Others	–	–	7.76	28
Mother's education				
No education	–	–	10.25	37
Primary	–	–	47.09	170
Secondary and above	–	–	42.66	154
Mother's occupation				
Business	–	–	1.11	4
Day labor	–	–	1.39	5
Garments worker	–	–	6.93	25
Homemaker	–	–	89.75	324
Others	–	–	0.83	3
Household income				
5000–10,000	–	–	34.35	124
11,000–15,000	–	–	36.01	130
16,000–20,000	–	–	18.01	65
20,000−+	–	–	11.63	42

### 3.2 Father's involvement in IYCF

Regarding fathers' involvement, overall, 63% (*n* = 229) of fathers were found to be categorized as good involvement, while 37% (*n* = 132) of them were labeled as poorly involved based on the scoring described in Section 2.

### 3.3 Factors affecting Fathers' involvement in IYCF

Fathers' involvement in IYCF practice was further investigated to find out whether there is any association with socioeconomic demographic variables. Mother's age (*p* = .049), mothers' education (*p* < .01), father's education (*p* < .01), and father's occupation (*p* < .01) were separately associated with involvement status. Table 2 shows the detailed results of the bivariate analysis.

**TABLE 2:** Bivariate analysis of background factors in IYCF practice.

Variable	Good involvement		Poor involvement		*p*‐Value
	%	*N*	%	*N*	
Child's age					
0–6 months	58	92	42	66	.077^a^
7–13 months	64	78	36	122	
14–20 months	77	47	23	14	
21–24 months	60	12	40	8	
Child's sex					
Male	60	105	40	71	.146^a^
Female	67	127	33	61	
Child's parity					
1st parity	68	111	32	53	.126^a^
Other than 1st parity	60	118	40	79	
Father's age					
19–30 years	63	130	37	75	.900
31–40 years	64	86	36	48	
≥41 years	59	59	41	9	
Father's education					
No education	38	19	62	31	.00^c^
Primary	61	98	39	62	
Secondary and above	74	112	26	39	
Father's occupation					
Business	72	39	28	15	.004^b^
Day labor	48	56	52	61	
Driver	60	6	40	4	
Garments worker	73	60	27	22	
Service	63	19	37	11	
Shop	78	25	22	7	
Unemployed	75	6	25	2	
Others	64	18	36	10	
Mother's age					
16–20 years	59	61	41	43	.049^b^
21–30 years	69	137	31	62	
31–40 years	53	31	47	27	
Mother's education					
No Education	35	13	65	24	.00^c^
Primary	61	104	39	66	
Secondary and above	73	112	27	42	
Mother's occupation					
Business	25	1	75	3	.33
Day labor	40	2	60	3	
Garments worker	72	18	28	7	
Homemaker	64	206	36	118	
Others	68	2	32	1	
Household income					
5000–10,000	61	76	39	48	.688
11,000–15,000	64	83	36	47	
16,000–20,000	69	45	31	20	
20,000–+	60	25	40	17	

^a^ Considered for multivariate analysis.

^b^ Significant <0.05.

^c^ Highly significant <0.01.

To observe the adjusted results, we included child's age, child's sex, child's parity, mothers' age, mother's education, father's education, and fathers' occupation in the multivariate model. It was observed that fathers were having about two times higher involvement in IYCF practice for their first child compared to the other children (aOR: 1.99, 95% CI = 1.04, 3.79, *p* = .03). Besides, those who had 14–20 months aged children were about three times more likely to have good involvement compared to those who had 0–6 months aged children (aOR = 2.73, 95% CI = 1.32, 5.64, *p* = <.01).

Those fathers whose wives were aged 21–30 years were two times more likely to have good involvement in IYCF practice compared to those whose wives were aged 16–20 (aOR = 2.34, 95% CI = 1.20, 4.58, *p* = .01). Fathers who attended primary education were 2.55 times (aOR = 2.55, 95% CI = 1.28, 5.11, *p* = .00) more likely to have good involvement in IYCF practice compared to those who did not attend any institutional education. A similar trend is also observed in fathers with secondary/higher secondary education (aOR = 3.98, 95% CI = 1.91, 8.26, *p* < .00).

On the other hand, day laborer fathers are 0.66% less likely (aOR = 0.34, 95% CI=, 0.16, 0.70, *p* = .00) to have good involvement than the business owner fathers. Table 3 shows the detailed results of multivariate analysis.

**TABLE 3:** Multivariate analysis of father's involvement in IYCF practice.

Variable	OR (95% CI)	*p*‐Value	aOR (95% CI)	*p*‐Value
Child's age				
0–6 months	Ref	Ref	Ref	Ref
7–13 months	1.27 (0.78–2.06)	.33	1.53 (0.89–2.64)	.12
14–20 months	2.40 (1.22–4.73)	.01	2.73 (1.32–5.64)	<.01
21–24 months	1.07 (0.41–2.77)	.88	1.16 (0.40–3.33)	.78
Child's sex				
Male	Ref	Ref		
Female	1.37 (0.89–2.11)	.14		
Child's parity				
1st parity	1.40 (0.90–2.16)	.12	1.99 (1.04–3.79)	.03
Other than 1st parity	Ref	Ref	Ref	Ref
Father's education				
No education	Ref		Ref	Ref
Primary	2.57 (1.34–4.95)	.00	2.55 (1.28–5.11)	<.01
Secondary and above	4.68 (2.37–9.22)	.00	3.98 (1.91–8.26)	<.01
Father's occupation				
Business	Ref	Ref	Ref	Ref
Day labor	0.35 (0.17–0.70)	.00	0.34 (0.16–0.70)	<.01
Driver	0.57 (0.14–2.33)	.44	0.50 (0.11–2.21)	.36
Garments worker	1.04 (0.48–2.26)	.90	0.82 (0.36–1.86)	.63
Service	0.66 (0.25–1.72)	.40	0.51 (0.18–1.42)	.20
Shop	1.37 (0.49–3.84)	.54	1.03 (0.35–3.03)	.94
Unemployed	1.15 (0.20–6.36)	.87	1.27 (0.21–7.67)	.79
Others	0.69 (0.26–1.83)	.46	0.54 (0.19–1.55)	.26
Mother's age				
16–20 years	Ref	Ref	Ref	Ref
21–30 years	1.55 (0.95–2.54)	.07	2.34 (1.20–4.58)	.01
31–40 years	0.80 (0.42–1.54)	.52	1.63 (0.67–3.99)	.27
Mother's education				
No education	Ref	Ref		
Primary	2.90 (1.38–6.11)	.00		
Secondary and above	4.92 (2.29–10.55)	.00		

## 4 DISCUSSION

The importance of the involvement of fathers during the complementary feeding period has been underscored across the globe (Bhutta et al., 2013). This research is one of the first studies that provides evidence of the present status of fathers' involvement in urban Bangladesh specifically focusing on the poor living in the slums. The study explored the proportion and associated factors of fathers' involvement in IYCF practices among urban slum dwellers in Bangladesh. It has been observed that the overall 63% of fathers had good involvement in IYCF practices. This is somewhat similar to a study conducted by Abera et al. in Ethiopia in 2017, which reported overall 72.4% involvement of fathers in breastfeeding practices (Abera et al., 2017). The existing literature suggests that fathers play a pivotal role in terms of ensuring child health and survival by providing support and encouragement to the mothers that can strengthen maternal confidence. Especially in terms of breastfeeding, mothers, who are supported by the child's father, were more likely to breastfeed their children for a longer duration (Mannion et al., 2013). Another recent study by Mithra et al. (2021) in India found that almost 41% of fathers had poor involvement in IYCF practices. Considering the geographic condition and sociodemographic profile, this study also has shown similar results where 37% of fathers were observed to have poor involvement in IYCF practice.

The finding suggests that fathers' occupation has a significant association with fathers' involvement in IYCF. This finding corroborates with other similar studies in Bangladesh. One previous research suggests that one of the important factors that is closely associated with the initiation of complementary foods at the recommended age is the occupation of the father (Kabir et al., 2012). This study revealed that the children whose fathers are engaged in occupations like rickshaw‐pulling or small enterprises had an increased risk of not starting complementary feeding in comparison with the fathers engaged in agricultural occupation. Besides, the households with fathers involved in occupations related to agriculture had better food security than the households where fathers are engaged in nonagricultural wage‐dependent occupations. This increased level of food security at the household level is linked with better infant feeding practices in Bangladesh (Kabir et al., 2012). Apart from food security issue, there is also a workplace time dimension of fathers' occupation that influence fathers' involvement in IYCF. For example, a study by Mallan et al. (2014) showed that fathers' time provided at the workplace adversely affects fathers' involvement in the child‐feeding practice. The finding from this study also suggests that day‐laborer fathers have less opportunity of having good involvement in IYCF practice compared to fathers engaged in business. It is plausible that day‐laborer fathers need to spend more time in their work, which impedes getting involved in the IYCF or caring practices. A recent systematic review on complementary feeding practices among Bangladeshi infants and young children also suggested that father's occupation is an influencing factor for complementary child‐feeding practices (Manikam et al., 2017).

Apart from fathers' occupation, fathers' education was significantly associated with father's involvement in the IYCF practice. It was observed that the more the fathers are educated, there is more chance of fathers' good involvement. A study conducted in Aceh, Indonesia by Ahmad et al. (2018) also has suggested that the father's education is significantly associated with child's nutrition and complementary feeding practices. Besides, the finding of this study is also congruent with the study conducted by The South Asia Infant Feeding Research Network (SAIFRN) which carried out analyses of existing demographic health survey (DHS) data to assess complementary feeding practices across countries in South Asia. The SAIFRN study revealed that low levels of parental education, especially fathers' education, were consistently associated with poor complementary feeding across South Asian countries (Senarath & Dibley, 2012).

The finding of this study did not reveal significant association between fathers' involvement in IYCF and monthly family income of the household. This finding does not match with other similar studies. A study conducted in Madagascar by Rakotomanana et al. (2017) identified that poor household wealth is associated with poor IYCF practices. The systematic review of Bangladeshi studies revealed that household income is a promoter of IYCF practices (Manikam et al., 2017). However, another study from West Bengal, India reported that although mothers' perception of IYCF was better among higher income households group, lowest practice was observed among this group (Das et al., 2013). However, finding from this study did not get any such association. Besides, the study finding suggests that there was no statistically significant association between fathers' involvement in IYCF and the sex of the child. This finding is also not aligned with other relevant researches. For example, a study conducted in the coastal region of southern India reported that the fathers of male children were more likely to have poor involvement in IYCF practices compared to those fathers who have female children (Mithra et al., 2021). Similarly, there is speculation from previous studies that a parent's age has the potential to influence his/her time with children. This is because, the younger the parents, the more time they might need to invest in career development, while the older ones might be more secure in their profession allowing them to contribute more to child care (Raley et al., 2012). However, the results of this study did not suggest significant association between father's age and fathers' involvement in IYCF practices.

Regarding child's parity, in this study, first‐time fathers have more likelihood to have good involvement in IYCF compared to their experienced counterparts. This finding is similar to another study conducted in India (Das et al., 2013). Furthermore, the study results showed that the fathers of children aged between 14 and 20 months had 2.82 times more chances of good involvement in IYCF practice compared to fathers of children aged 0–6 months of age. This result is congruent with other similar studies that showed fathers take comparatively reduced role in feeding babies at the early stage, but become more responsible in child feeding during the preschool years (Baxter & Smart, 2011; Daniels et al., 2009). A similar study conducted by Tikotzky et al. (2010) in Israel revealed that fathers' involvement was significantly lower in terms of nighttime baby care at an early age.

The limitation of the study is that the emphasis was primarily provided only toward fathers' actual involvement, and details of the other sociodemographic variables like access to media and possession of wealth were not included, which could be an influencing factor in the fathers' involvement in IYCF practices. Furthermore, the study explored the father's involvement from mothers' perspective using a mother‐report questionnaire. It was done to avoid the social desirability bias. However, there are still chances of overestimating and underestimating the fathers' involvement status, which should be considered as an additional limitation of the study. Besides, since this is a cross‐sectional study, it gives only a snapshot of the situation without drawing any causal relationship among variables. The strength of the study is that the topic targeted had been the comparatively neglected, yet useful, of fathers' involvement in IYCF practice in Bangladesh. Considering the dearth of policy attention provided to the urban poor, this research, therefore, adds important insights into the current status of fathers' involvement and future programmatic directions. The government of Bangladesh has formulated a national strategy on IYCF with minimal focus on engaging fathers in the whole process. As government programs are aiming for overcoming malnutrition of mothers and children and its negative health consequences across all regions of the country, this study tried to address the scarcity of information on to what extent fathers' involvement is prevalent among the urban poor people. This study has comparatively small sample size considering the resources available. Therefore, more research is needed in this context to properly implement the guideline. Fathers' involvement is instrumental in harnessing positive effect on child's nutritional and cognitive outcome. The finding of the study has the potential to inform program planners about the current status of fathers' involvement in IYCF practices in urban slum context of Bangladesh. The finding can help the planners to think about the extent of necessity to engage the fathers in future IYCF programs for urban slums. In addition, the study finding elucidated factors associated with fathers' involvement, which can also be helpful for designing future interventions. For example, the finding suggests that fathers' education was positively associated with father's involvement status. Therefore, future interventions targeting to reduce child malnutrition in urban slums can include specific educational awareness programs for fathers living in urban slums of Bangladesh.

## 5 CONCLUSION

The study finding showed that more than half of the fathers had good involvement in IYCF practice and child care in selected slum areas in Bangladesh. Fathers' education and occupation were the factors that influenced the status of fathers' involvement. Besides, mothers' age and education were also influencing fathers' involvement in the IYCF practices. The child's age was also found toward significant.

However, more than one third of the (35%) fathers had poor involvement. The study results will be helpful to assess the proportion and associated factors of fathers' involvement in IYCF practices among urban slum dwellers in Bangladesh. Further mixed‐methods and longitudinal studies are recommended to be undertaken to delve into the deeper contextual issues and establish causal relationship between estimates. Besides, the study finding might be helpful for developing new programs or interventions in improving the nutrition status of children in the urban slums of Bangladesh. From the study findings, we recommend that particular community‐based programs should be taken targeting fathers to sustain and improve fathers' involvement in IYCF and child‐rearing practice in most vulnerable groups.

## AUTHOR CONTRIBUTIONS

**Dipika Shankar Bhattacharyya:** Conceptualization (equal); data curation (lead); formal analysis (lead); investigation (lead); methodology (equal); project administration (lead); supervision (lead); writing – original draft (lead); writing – review and editing (lead). **Tonmoy Sarker:** Formal analysis (equal); methodology (supporting); writing – original draft (supporting); writing – review and editing (supporting). **Nargis Akter:** Data curation (equal); project administration (equal); supervision (lead); writing – original draft (supporting). **Sohana Shafique:** Methodology (supporting); writing – original draft (supporting); writing – review and editing (equal). **Mohammad Hayatun Nabi:** Conceptualization (supporting); methodology (supporting); writing – original draft (supporting); writing – review and editing (supporting). **Mohammad Delwer Hossain Hawlader:** Conceptualization (supporting); methodology (supporting); writing – original draft (supporting); writing – review and editing (supporting). **Dipak Kumar Mitra:** Conceptualization (lead); methodology (equal); supervision (equal); validation (equal); writing – original draft (equal); writing – review and editing (equal).

## FUNDING INFORMATION

The authors have not received any funding for this work.

## CONFLICT OF INTEREST STATEMENT

The authors declare no competing interests.

## Data Availability

The data that support the findings of this study are available from the corresponding author upon reasonable request.

## References

[fsn33390-bib-0001] Abera, M. , Abdulahi, M. , & Wakayo, T. (2017). Fathers' involvement in breast feeding practices and associated factors among households having children less than six months in Southern Ethiopia: A cross sectional study. Pediatrics & Therapeutics, 7(1), 1000306.

[fsn33390-bib-0002] Adams, A. M. , Ahmed, S. M. , & Evans, T. G. (2018). Universal health care in Bangladesh—Promises and perils. The Lancet Global Health, 6(1), e10–e11. 29241601 10.1016/S2214-109X(17)30470-9

[fsn33390-bib-0003] Adams, A. M. , Islam, R. , & Ahmed, T. (2015). Who serves the urban poor? A geospatial and descriptive analysis of health services in slum settlements in Dhaka, Bangladesh. Health Policy and Planning, 30(suppl_1), i32–i45. 25759453 10.1093/heapol/czu094 PMC4353891

[fsn33390-bib-0004] Ahmad, A. , Madanijah, S. , Dwiriani, C. M. , & Kolopaking, R. (2018). Complementary feeding practices and nutritional status of children 6–23 months old: Formative study in Aceh, Indonesia. Nutrition Research and Practice, 12(6), 512–520. 30515279 10.4162/nrp.2018.12.6.512 PMC6277313

[fsn33390-bib-0005] Baxter, J. , & Smart, D. (2011). Fathering in Australia among couple families with young children. *Australian Department of Families, Housing, Community Services and Indigenous Affairs, Occasional Paper* (37).

[fsn33390-bib-0006] Bhutta, Z. A. , Das, J. K. , Rizvi, A. , Gaffey, M. F. , Walker, N. , Horton, S. , Webb, P. , Lartey, A. , Black, R. E. , Lancet Nutrition Interventions Review Group , & the Maternal and Child Nutrition Study Group . (2013). Evidence‐based interventions for improvement of maternal and child nutrition: What can be done and at what cost? The Lancet, 382(9890), 452–477. 10.1016/S0140-6736(13)60996-4 23746776

[fsn33390-bib-0007] Black, R. E. , Victora, C. G. , Walker, S. P. , Bhutta, Z. A. , Christian, P. , De Onis, M. , Ezzati, M. , Grantham‐McGregor, S. , Katz, J. , Martorell, R. , Uauy, R. , & Maternal and Child Nutrition Study Group . (2013). Maternal and child undernutrition and overweight in low‐income and middle‐income countries. The Lancet, 382(9890), 427–451. 10.1016/S0140-6736(13)60937-X 23746772

[fsn33390-bib-0008] Daniels, L. A. , Magarey, A. , Battistutta, D. , Nicholson, J. M. , Farrell, A. , Davidson, G. , & Cleghorn, G. (2009). The NOURISH randomised control trial: Positive feeding practices and food preferences in early childhood‐a primary prevention program for childhood obesity. BMC Public Health, 9(1), 1–10. 19825193 10.1186/1471-2458-9-387 PMC2770488

[fsn33390-bib-0009] Das, N. , Chattopadhyay, D. , Chakraborty, S. , & Dasgupta, A. (2013). Infant and young child feeding perceptions and practices among mothers in a rural area of West Bengal, India. Annals of Medical and Health Sciences Research, 3(3), 370–375. 24116316 10.4103/2141-9248.117955 PMC3793442

[fsn33390-bib-0010] Directorate General of Health Services . (2011). Operational Plan For “National Nutrition Services”, July 2011–June 2016, Health, Population and Nutrition Sector Development Program (HPNSDP) . https://www.unicef.org/bangladesh/sites/unicef.org.bangladesh/files/2018‐10/NNS%20OP%202011‐2016.pdf

[fsn33390-bib-0011] Fan, X. , & Chen, M. (2001). Parental involvement and students' academic achievement: A meta‐analysis. Educational Psychology Review, 13(1), 1–22.

[fsn33390-bib-0012] Grantham‐McGregor, S. (2007). Early child development in developing countries. The Lancet, 369(9564), 824. 10.1016/S0140-6736(07)60404-8 17350449

[fsn33390-bib-0013] Hackett, K. M. , Mukta, U. S. , Jalal, C. S. , & Sellen, D. W. (2015). A qualitative study exploring perceived barriers to infant feeding and caregiving among adolescent girls and young women in rural Bangladesh. BMC Public Health, 15(1), 1–11. 26259575 10.1186/s12889-015-2115-5 PMC4531479

[fsn33390-bib-0014] Haider, R. , Rasheed, S. , Sanghvi, T. G. , Hassan, N. , Pachon, H. , Islam, S. , & Jalal, C. S. (2010). Breastfeeding in infancy: Identifying the program‐relevant issues in Bangladesh. International Breastfeeding Journal, 5(1), 1–12. 21118488 10.1186/1746-4358-5-21 PMC3009955

[fsn33390-bib-0015] Hume‐Nixon, M. , & Kuper, H. (2018). The association between malnutrition and childhood disability in low‐and middle‐income countries: Systematic review and meta‐analysis of observational studies. Tropical Medicine & International Health, 23(11), 1158–1175. 30151939 10.1111/tmi.13139

[fsn33390-bib-0016] Institute of Public Health Nutrition . (2007). National Strategy for infant and young child feeding in Bangladesh. Directorate General of Health Services Ministry of Health and Family Welfare Government of the People's Republic of Bangladesh. http://etoolkits.dghs.gov.bd/sites/default/files/national_strategy_for_iycf.pdf

[fsn33390-bib-0017] Jeynes, W. H. (2007). The relationship between parental involvement and urban secondary school student academic achievement: A meta‐analysis. Urban Education, 42(1), 82–110.

[fsn33390-bib-0018] Joshi, N. , Agho, K. E. , Dibley, M. J. , Senarath, U. , & Tiwari, K. (2012). Determinants of inappropriate complementary feeding practices in young children in Nepal: Secondary data analysis of Demographic and Health Survey 2006. Maternal & Child Nutrition, 8, 45–59. 22168518 10.1111/j.1740-8709.2011.00384.x PMC6860874

[fsn33390-bib-0019] Kabir, I. , Khanam, M. , Agho, K. E. , Mihrshahi, S. , Dibley, M. J. , & Roy, S. K. (2012). Determinants of inappropriate complementary feeding practices in infant and young children in Bangladesh: Secondary data analysis of Demographic Health Survey 2007. Maternal & Child Nutrition, 8, 11–27. 22168516 10.1111/j.1740-8709.2011.00379.x PMC6860519

[fsn33390-bib-0020] Lata, L. N. (2020). Neoliberal urbanity and the right to housing of the urban poor in Dhaka, Bangladesh. Environment and Urbanization ASIA, 11(2), 218–230.

[fsn33390-bib-0021] Mallan, K. , Nothard, M. , Thorpe, K. , Nicholson, J. , Wilson, A. , Scuffham, P. , & Daniels, L. (2014). The role of fathers in child feeding: Perceived responsibility and predictors of participation. Child: Care, Health and Development, 40(5), 715–722. 23902382 10.1111/cch.12088

[fsn33390-bib-0022] Manikam, L. , Robinson, A. , Kuah, J. Y. , Vaidya, H. J. , Alexander, E. C. , Miller, G. W. , Singh, K. K. , Dawe, V. , Ahmed, S. , Lingam, R. , & Lakhanpaul, M. (2017). A systematic review of complementary feeding practices in South Asian infants and young children: The Bangladesh perspective. BMC Nutrition, 3(1), 1–13. 10.1186/s40795-017-0176-9 PMC7050712 32153836

[fsn33390-bib-0023] Mannion, C. A. , Hobbs, A. J. , McDonald, S. W. , & Tough, S. C. (2013). Maternal perceptions of partner support during breastfeeding. International Breastfeeding Journal, 8(1), 1–7. 23651688 10.1186/1746-4358-8-4 PMC3653816

[fsn33390-bib-0024] McGuire, S. (2015). World Health Organization. Comprehensive implementation plan on maternal, infant, and young child nutrition. Geneva, Switzerland, 2014. Advances in Nutrition, 6(1), 134–135. 25593153 10.3945/an.114.007781 PMC4288273

[fsn33390-bib-0025] Mithra, P. , Bhaskaran Unnikrishnan, R. T. , Kumar, N. , Holla, R. , & Rathi, P. (2021). Paternal involvement in and sociodemographic correlates of infant and young child feeding in a district in coastal South India: A cross‐sectional study. Frontiers in Public Health, 9, 661058. 34150705 10.3389/fpubh.2021.661058 PMC8212972

[fsn33390-bib-0026] NIPORT , icddr, b , & Evaluation, M . (2015). Bangladesh urban health survey . https://www.measureevaluation.org/publications/tr‐15‐117.html

[fsn33390-bib-0027] Prentice, A. M. , Ward, K. A. , Goldberg, G. R. , Jarjou, L. M. , Moore, S. E. , Fulford, A. J. , & Prentice, A. (2013). Critical windows for nutritional interventions against stunting. The American of Clinical Nutrition, 97(5), 911–918. 10.3945/ajcn.112.052332 PMC3628381 23553163

[fsn33390-bib-0028] Rakotomanana, H. , Gates, G. E. , Hildebrand, D. , & Stoecker, B. J. (2017). Situation and determinants of the infant and young child feeding (IYCF) indicators in Madagascar: Analysis of the 2009 Demographic And Health Survey. BMC Public Health, 17(1), 1–9. 29037229 10.1186/s12889-017-4835-1 PMC5644246

[fsn33390-bib-0029] Raley, S. , Bianchi, S. M. , & Wang, W. (2012). When do fathers care? Mothers' economic contribution and fathers' involvement in child care. American Journal of Sociology, 117(5), 1422–1459. 10.1086/663354 PMC4568757 26379287

[fsn33390-bib-0030] Ruel, M. T. , Levin, C. E. , Armar‐Klemesu, M. , Maxwell, D. , & Morris, S. S. (1999). Good care practices can mitigate the negative effects of poverty and low maternal schooling on children's nutritional status: Evidence from Accra. World Development, 27(11), 1993–2009.

[fsn33390-bib-0031] Sanghvi, T. , Martin, L. , Hajeebhoy, N. , Abrha, T. H. , Abebe, Y. , Haque, R. , Tran, H. T. T. , & Roy, S. (2013). Strengthening systems to support mothers in infant and young child feeding at scale. Food and Nutrition Bulletin, 34(3_suppl2), S156–S168. 24261074 10.1177/15648265130343S203

[fsn33390-bib-0032] santé, O. M. D. I. , Organization, W. H , Staff, W. H. O. , UNICEF , l'enfance, F. D. N. U. P. , & UNAIDS . (2003). Global strategy for infant and young child feeding. World Health Organization.

[fsn33390-bib-0033] Sarkadi, A. , Kristiansson, R. , Oberklaid, F. , & Bremberg, S. (2008). Fathers' involvement and children's developmental outcomes: A systematic review of longitudinal studies. Acta Paediatrica, 97(2), 153–158. 18052995 10.1111/j.1651-2227.2007.00572.x

[fsn33390-bib-0034] Senarath, U. , Agho, K. E. , Akram, D. E. S. , Godakandage, S. S. , Hazir, T. , Jayawickrama, H. , Joshi, N. , Kabir, I. , Khanam, M. , Patel, A. , Pusdekar, Y. , Roy, S. K. , Siriwardena, I. , Tiwari, K. , & Patel, A. (2012). Comparisons of complementary feeding indicators and associated factors in children aged 6–23 months across five South Asian countries. Maternal & Child Nutrition, 8, 89–106. 22168521 10.1111/j.1740-8709.2011.00370.x PMC6860856

[fsn33390-bib-0035] Senarath, U. , & Dibley, M. J. (2012). Complementary feeding practices in South Asia: Analyses of recent national survey data by the South Asia Infant Feeding Research Network. Maternal & Child Nutrition, 8, 5–10. 22168515 10.1111/j.1740-8709.2011.00371.x PMC6860754

[fsn33390-bib-0036] Shafique, S. , Bhattacharyya, D. S. , Anwar, I. , & Adams, A. (2018). Right to health and social justice in Bangladesh: Ethical dilemmas and obligations of state and non‐state actors to ensure health for urban poor. BMC Medical Ethics, 19(1), 61–69. 29945594 10.1186/s12910-018-0285-2 PMC6019983

[fsn33390-bib-0037] Tandon, P. S. , Tovar, A. , Jayasuriya, A. T. , Welker, E. , Schober, D. J. , Copeland, K. , Dev, D. A. , Murriel, A. L. , Amso, D. , & Ward, D. S. (2016). The relationship between physical activity and diet and young children's cognitive development: A systematic review. Preventive Medicine Reports, 3, 379–390. 27419040 10.1016/j.pmedr.2016.04.003 PMC4929214

[fsn33390-bib-0038] Tikotzky, L. , Sadeh, A. , & Glickman‐Gavrieli, T. (2010). Infant sleep and paternal involvement in infant caregiving during the first 6 months of life. Journal of Pediatric Psychology, 36(1), 36–46. 20444853 10.1093/jpepsy/jsq036

[fsn33390-bib-0039] Tuthill, E. , McGrath, J. , & Young, S. (2014). Commonalities and differences in infant feeding attitudes and practices in the context of HIV in sub‐Saharan Africa: A metasynthesis. AIDS Care, 26(2), 214–225. 23879637 10.1080/09540121.2013.813625 PMC3855184

[fsn33390-bib-0040] UNICEF , & WHO, W . (2020). Levels and trends in child malnutrition: Key findings of the 2019 edition of the Joint Child Malnutrition Estimates. World Health Organization.

